# Psychometric Properties of the Perceived Collective Family Efficacy Scale in Algeria

**DOI:** 10.3390/healthcare11192691

**Published:** 2023-10-08

**Authors:** Aiche Sabah, Musheer A. Aljaberi, Kuo-Hsin Lee, Chung-Ying Lin

**Affiliations:** 1 Faculty of Human and Social Sciences, Hassiba Benbouali University of Chlef, Chlef 02076, Algeria; 2 Faculty of Medicine and Health Sciences, Taiz University, Taiz 6803, Yemen; musheer.jaberi@gmail.com; 3 Department of Emergency Medicine, E-Da Dachang Hospital, I-Shou University, Kaohsiung 824, Taiwan; 4 School of Medicine, College of Medicine, I-Shou University, No. 8, Yi-Da Road, Jiao-Su Village, Yan-Chao District, Kaohsiung 824, Taiwan; 5 Institute of Allied Health Sciences, College of Medicine, National Cheng Kung University, Tainan 701, Taiwan; cylin36933@gmail.com

**Keywords:** self-efficacy, collective efficacy, psychological theory, family, factor analysis, Rasch model, scales

## Abstract

The Perceived Collective Family Efficacy Scale is a tool utilized to assess the effectiveness of a family as a functioning system. The scale has a single-factor structure with good validity and reliability. However, there is a shortage of psychometric evidence of the scale in an Arab context. This study aimed to assess the psychometric properties of the Perceived Collective Family Efficacy Scale among Algerian students. A cross-sectional study was conducted to recruit 300 students from Algerian universities. The students completed the 20-item Perceived Collective Family Efficacy Scale, Arabic version, to measure their beliefs regarding collective efficacy within families. Confirmatory factor analysis (CFA) and the Rasch model were employed to assess the psychometric properties and unidimensionality of the scale. Both CFA and Rasch findings supported the single-factor structure for the Perceived Collective Family Efficacy Scale. Specifically, the CFA indicated that the data aligned with a one-dimensional model. The Rasch analysis revealed favorable indicators of unidimensionality for the scale. Moreover, a thorough examination of the Principal Component Analysis of the Rasch residuals confirmed the existence of a single dimension, which is consistent with the original structure of the Perceived Collective Family Efficacy Scale. These findings provide scientific evidence for the validity and unidimensional nature of the Perceived Collective Family Efficacy Scale. Specifically, the satisfactory psychometric properties findings indicate that the Perceived Collective Family Efficacy Scale could be applied in an Arab context (i.e., in Algerian). The scale’s unidimensional structure underscores its effectiveness in measuring beliefs in collective efficacy within families. These results enhance our understanding of family dynamics and provide a reliable measurement tool for assessing family efficacy in similar cultural contexts.

## 1. Introduction

Family provides more than environments where individuals live; it also provides a complete and intricate social system for human development. Individuals interact within these systems, influencing each other’s behavior. As a social system, the family is envisioned to possess unique characteristics, rules, roles, communication patterns, and power structures that extend beyond the individual [1,2,3,4]. The family systems theory asserts that family subsystems are closely interconnected, conceptualizing families as organized groups. It also suggests that understanding human behavior relies on the interactions between individuals within the family and between the family and its context, as the family is an integral part of its surrounding environment [5,6,7]. According to the family systems theory, family functioning encompasses task accomplishment, role performance, emotional involvement, control, values, standards, expression, and emotional communication. The concept of family functioning includes both the efficiency and style of the family. Family efficiency requires structure and the ability to adapt to changes over time, while family patterns refer to the quality of family interactions [8,9].

The social cognitive theory links behavior to four factors: goals, outcome expectations, self-efficacy, and social–structural variables [10,11,12]. The social cognitive theory, as proposed by Bandura, assumes an interaction between personal, behavioral, and social–environmental factors. The key point is that people strive to develop a sense of significant control over important events in their lives. The perceived efficacy of the group influences their aspirations, resource utilization, contribution to collective effort, resilience in the face of failed collective efforts or opposition, and adaptability when confronting challenging problems. Thus, the social cognitive theory establishes a central role for perceived efficacy in managing various relationships, interactions, and daily tasks within the family system [13,14]. Specifically, collective efficacy beliefs within the family refer to the judgments made by family members regarding the family’s collective ability to accomplish necessary tasks for its functioning. Family collective efficacy focuses on the capabilities of family members to work together as a whole [15]. In order to better understand family collective efficacy, a validated instrument (i.e., the Perceived Collective Family Efficacy Scale in this study) should be used. However, the Perceived Collective Family Efficacy Scale does not have an Arabic version to assess Arab populations. Therefore, the present study translated the Collective Family Efficacy Scale into Arabic for further psychometric evaluation.

### 1.1. Collective Efficacy in Families

Self-efficacy is the belief in one’s ability to perform complex life tasks successfully. It plays a crucial role in shaping a person’s feelings, perceptions, motivational activities, and behaviors across various activities [16]. Collective efficacy is considered an extension of building self-efficacy and is a subsidiary model of the social cognitive theory proposed by Bandura. Bandura [17] defines collective efficacy as “a group’s shared belief in its conjoint capabilities to organize and execute the actions required to produce given levels of attainment”. Thus, perceived collective efficacy within the organization represents the group members’ beliefs regarding the collective ability of the social system [18,19]. The dynamic characteristics of a group can encompass social support, solidarity, communication, collective participation, dialogue, trust, decision-making and sharing, group belongingness, and common goals. The willingness and ability to intervene for the benefit of the group depend on the level of solidarity, participation, and mutual trust among group members [20,21]. Beliefs about collective family efficacy reflect the judgments made by family members regarding the collective ability of the family as a whole to function as a complete system in accomplishing necessary tasks for the functioning of the family. Bandura, et al. [22] define perceived collective family efficacy as: “members’ beliefs in the capabilities of their family to work together to promote each other’s development and well-being, maintain beneficial ties to extrafamilial systems, and exhibit resilience to adversity”.

While other self-efficacy beliefs primarily focus on dyadic relationships (e.g., between parent and child, husband and wife), collective family efficacy beliefs center around the perceived practical capabilities of the family as a whole [23]. Individual self-efficacy beliefs alone may be insufficient to achieve desired goals when focusing on family performance. Spouses, parents, and children cannot fulfill their roles independently of other family members’ feelings, expectations, and behaviors. Many outcomes can only be achieved when all family members pool their resources and efforts together. This is because the family, as a social system, has a lasting impact on individual growth. Individuals face a variety of needs and challenges throughout life as part of an interconnected family system. Similar to any other social system, perceived collective efficacy influences the system’s sense of purpose and message, the strength of members’ commitment to its pursuit, their perception of their ability to fulfill mutual obligations, and the family’s resilience in the face of adversity [17,23,24]. According to a study by Bandura, Caprara, Barbaranelli, Regalia and Scabini [22], a high sense of collective family efficacy is associated with open family communication and explicit disclosure by teenagers about their activities outside the home. Furthermore, family collective efficacy has contributed to the satisfaction of parents and teenagers with their family life. Another study by Kao, et al. [25] found that the perceived collective family efficacy of both teenagers and parents reduced the impact of parental and teenage depressive symptoms on risky health behaviors among teenagers. In fact, parents’ and teenagers’ perceived collective family efficacy protects against depressive symptoms and risky health behaviors.

### 1.2. Perceived Collective Family Efficacy Scale

The Perceived Collective Family Efficacy Scale, developed by Caprara [26] and Caprara, Regalia, Scabini, Barbaranelli and Bandura [24], is a measure used to assess the perceived effectiveness of families in accomplishing essential tasks and functioning as a complete system. It focuses on the family’s practical capabilities and views it as a social system comprising interconnected and interactive relationships. This scale comprises 20 items that emphasize the family’s ability to manage daily routines, reach consensus in decision-making and planning, cope with challenges, promote mutual agreement, provide emotional support during difficult times, engage in shared activities and relaxation despite multiple commitments, and maintain positive relationships with the community.

Several studies have been conducted on the Perceived Collective Family Efficacy Scale’s psychometric properties. Caprara, Regalia, Scabini, Barbaranelli and Bandura [24] used a group of parents and adolescents to validate the scale’s reliability and validity. The Principal Component Analysis (PCA) with oblimin rotation showed that collective family efficacy is unidimensional. The Cronbach’s alpha coefficient for the collective family scale indicated high internal consistency, with values of 0.96 for boys and 0.97 for girls. The correlation coefficients between parents’ and adolescents’ family efficacy beliefs ranged from low to moderately high congruence, with same-sex dyads typically having stronger correlations than opposite-sex dyads.

Costa and colleagues conducted studies in Portuguese and Italian contexts to validate the cross-cultural stability of the Perceived Collective Family Efficacy Scale and its associations with communication, conflict management, and children’s academic achievement [27]. The Perceived Collective Family Efficacy Scale’s factor loadings were found to be robust in both samples, ranging from 0.71 to 0.89 in Portugal and 0.71 to 0.89 in Italy, indicating that cross-cultural invariance had been achieved in terms of configurable, metric, and scalar. The construct validity was supported by various correlations with internalized and externalized symptoms, close communication with parents, aggressive conflict styles, open communication, compromise in conflict styles, and children’s academic achievement.

Pepe, et al. [28] conducted validation studies in Spanish adolescents and found that all items displayed factor loadings exceeding 0.40, indicating a robust relationship with the underlying factor. The Cronbach’s alpha coefficient achieved a value of 0.92, meeting the standard criteria for internal consistency. The construct validity was supported by various correlations, including positive correlations between perceived collective family efficacy and parental affection, the promotion of autonomy, and productive coping strategies and negative correlations with psychological control exerted by parents. The scale also exhibited positive correlations with certain non-productive coping strategies (e.g., worry, wishful thinking) and negative correlations with others (e.g., tension reduction). Additionally, adolescents with a higher family efficacy tended to use fewer drugs.

The psychometric properties of the Perceived Family Collective Efficacy revised scales were also evaluated in the Iranian population by Panaghi and colleagues [29]. Exploratory factor analysis revealed a two-factor solution, while confirmatory factor analysis provided support for both the two-factor and one-factor models, with a preference for the two-factor model due to its superior fit. The calculated Cronbach’s alpha coefficient was 0.92, and the test–retest reliability score was 0.83, highlighting high internal consistency and stability. These findings suggest that the Perceived Family Collective Efficacy Scale has robust psychometric properties suitable for research and family counseling endeavors within the Iranian context.

Overall, the literature evidence indicates that the Perceived Collective Family Efficacy Scale is reliable and valid in assessing family effectiveness and functioning. Its utility spans different cultural contexts and age groups, making it a valuable tool for research and psychological assessment.

### 1.3. Purpose of the Present Study

The Perceived Collective Family Efficacy Scale has yet to be validated in Arab populations. Because of the lack of the Perceived Collective Family Efficacy Scale, studies conducted in Arabic could not investigate in-depth research on family collective efficacy. Introducing this new research tool in this field provides a consistent and reliable way to assess family efficacy, particularly in the quickly evolving social, economic, and political landscape of Arab societies, with a focus on Algeria. These changes significantly impact family life, social upbringing processes, marital harmony, and stability [30], including the psychological long-term effects of COVID-19 and quarantine, as well as future pandemics [4,31,32,33]. Given developing countries’ unique challenges, a trustworthy method for measuring family efficacy is crucial. The study examines the scale’s psychometric properties and expands its applicability to include various Arab cultures, making it a valuable addition to research tools for the wider Arab community.

The Perceived Collective Family Efficacy Scale has yet to be validated in Arab populations. Considering that the widespread use of this scale within families represents a valuable tool for assessing collective efficacy, this current study aims to verify the psychometric properties of the Perceived Collective Family Efficacy Scale among Algerian university students. Confirmatory factor analysis (CFA) and the Rasch model were the primary psychometric methods to assess the Perceived Collective Efficacy Scale’s psychometric properties. 

The objectives of this study are as follows: (1) To verify the psychometric properties of the translated and adapted version of the Perceived Collective Family Efficacy Scale through CFA on Algerian university students. (2) To examine the psychometric properties of the translated and adapted version of the Perceived Collective Family Efficacy Scale within the framework of Rasch modeling on Algerian university students.

## 2. Materials and Methods

### 2.1. Study Design and Participants

A cross-sectional research design was used in this study to create general models that relate groups of variables under specific conditions [34,35,36]. The data were manually collected through the Perceived Collective Family Efficacy Scale self-administered questionnaire to students from various disciplines at the University of Chlef. The response process was voluntary, and students were informed about the scale’s purpose, with participation being optional. Students who did not have siblings at home were excluded from the study. The participants in this study completed the Arabic-translated version of the Perceived Collective Family Efficacy Scale. The research was conducted as a part of the research conducted by Projects on University Training and Research (PRFU), Chlef University, and the research was approved by the Ministry of Higher Education and Scientific Research, Hassiba Benbouali University of Chlef, Algeria, Faculty of Humanities and Social Sciences, Department of Social Sciences with ethical approval reference number (I05L03UN020120200002).

### 2.2. Study Sample Size

Based on research by Hair et al., a sample size of at least 100 is necessary to conduct a structural equation modeling (SEM) analysis using covariance [37]. Given that confirmatory factor analysis (CFA) is a type of SEM, the sample size of 300 students in the present study was thus deemed suitable for utilizing CFA to achieve research objectives [38,39]. The importance of sample size in modeling and CFA studies has been emphasized by various researchers, with a consensus that SEM (including CFA) studies require a minimum of 200 participants [40,41]. However, it is worth noting that this recommended sample size may be insufficient for complex models, non-normal data distributions, or when using different estimation methods than maximum likelihood (ML), as Kline has pointed out [38,40]. Our study’s model is not complex as it only included one latent variable with 20 observed variables; therefore, we adopted the N: q rule introduced by Jackson [42,43]. With the use of 10:1 sample size ratio to the number of parameters to be estimated in SEM [40], the minimum sample size required is 200. In brief, a sample size of 200 is often considered reasonable for relatively small and simple models [44,45], such as the model tested in the present study.

### 2.3. Instrument

The Perceived Collective Family Efficacy was assessed using a 20-item scale developed by Caprara [26] to measure beliefs regarding the family’s effectiveness in functioning as a complete system and accomplishing essential tasks for family functioning. The scale covered various aspects of the family’s capabilities, including managing daily routines, reaching consensus in decision-making and planning, dealing with challenges, promoting mutual agreement, providing emotional support during difficult situations, enjoying and relaxing together despite multiple responsibilities, and maintaining positive relationships with the community as a whole. Participants rated each item on a 5-point scale ranging from 1 for “Not at all” to 5 for “Very well”. All items are positive, and there are no negative items. The scale spans from 20 to 100, with higher scores indicating a higher family self-efficacy. To calculate family self-efficacy, the mean of all items is computed. The scale also exhibits excellent internal consistency, with Cronbach’s alpha coefficients ranging from 0.96 to 0.97 [28].

#### Translation Procedures for the Scale

To translate the scale into Arabic, back-translation is the preferred translation technique. This method involves a group of interpreters and experts who translate the items from the source language to the target language and then back-translate them into the source language, ensuring agreement on meaning and word choice for each item [46,47]. Afterward, a small test group of participants is used to confirm that the target population easily understands the tool. To ensure cultural appropriateness, investigators should use commonly used words by the target population [48,49,50].

Therefore, the scale was translated from English to Arabic through a series of steps [45,46,51,52]. Firstly, permission was obtained from the developers (i.e., Caprara, Regalia, Scabini, Barbaranelli and Bandura [24]) to translate the scale from the English version (see Supplementary Materials, Table S1) [28] into Arabic.

A team of proficient Arab researchers and an English language expert with a good command of Arabic conducted a preliminary translation. The two translations were harmonized to create an initial Arabic scale version (Supplementary Materials, Table S2). During this step, a comparative analysis was conducted between the preliminary translation and the original scale to select clear vocabulary and phrases that are closely aligned with the English version.

In the second stage, a “Back Translation” process was carried out. An English language expert, who had not seen the scale’s English or Arabic versions, translated the proposed Arabic version back into English (Supplementary Materials, Table S3). The back-translated English version of the instrument was compared to the original English version, which increased confidence in the proposed Arabic translation. The comparison revealed a near-perfect match between the English translation and the original, particularly concerning the scale’s items. Some items varied slightly in wording but did not significantly impact their intended meaning. Notable item translations included:

*Item 1: “Set aside leisure time with your family when other things press for attention” became “Allocate free time for the family when there are other things that require attention.”*
*Item 3: “Resolve conflicts when family members feel they are not being treated fairly” became “Resolve conflicts when other people feel like they are not being treated fairly.”*
*Item 15: “Celebrate family traditions even in difficult times” became “Celebrate family occasions even during hard times.”*
*Item 17: “Face up to difficulties without excessive tension” became “Face difficulties effortlessly.”*

After confirming the accuracy of the back-translation, the scale was administered to a small sample of university students to ensure clarity of vocabulary, suitability of items, and comprehensibility of instructions for the target age group. It was found that the scale items were clear and free from ambiguity.

Following these steps, researchers were confident that the Arabic version of the questionnaire was ready for implementation. The scale was subsequently administered in Arabic to a sample of students at the University of Chlef.

### 2.4. Data Analysis

Descriptive statistics were used to summarize the item properties, providing information on central tendencies such as skewness, kurtosis, mean, and standard deviation. According to Hair Jr, et al. [53], skewness values between −1 and +1 are considered excellent, while kurtosis values should fall within the range of −2 to +2. These statistics offer a concise overview of the distribution and characteristics of the items in the scale [39,54,55].

To assess the factor structure of the Perceived Collective Family Efficacy Scale, CFA with the maximum likelihood estimation method was conducted to test if the scale has a single-factor structure. The following fit indices were used to examine data–model fit: the *p*-value of the chi-squared statistic (non-significant), comparative fit index (CFI) (≥0.90), standardized root-mean-squared residual (SRMR) (≤0.08), and root-mean-squared error of approximation (RMSEA) (≤0.08) [33,39,56,57,58,59]. By examining the relationship between the observed data and the expected factor structure, the CFA provided insights into how well the items were related to the measured latent construct [31,56,60,61,62]. In addition, Cronbach’s alpha, Composite Reliability, and MaxR(H) were used to estimate the scale’s internal reliability.

The Rasch model was also employed to confirm the unidimensionality of the Perceived Collective Family Efficacy Scale. Rasch analysis is a statistical method used to examine the properties of items (questions) and individuals on a measurement scale. It aims to assess the extent to which the items in a scale function together to measure a latent trait or construct accurately. The Rasch model is a widely used statistical model in psychometrics that assesses how well the observed responses align with the expected response patterns based on the underlying construct [56,63]. This analysis helps ensure that the scale is unidimensional, meaning that all items effectively contribute to measuring the intended construct. In Rasch analysis, we used Outfit mean square (MnSq) and Infit MnSq through Winsteps software version 3.72.3. The first step in Rasch analysis was to exclude individuals whose data did not fit the model, meaning their fit exceeded a threshold of 2. The acceptable fit range for individuals is typically between 0.60 and −1.40, as suggested by Bond and Fox [64]. Additionally, according to Linacre [65], several conditions should be considered when assessing the fit of individuals and items to the Rasch model. These conditions include examining Outfit before Infit prioritizing mean squares before ZSTD, prioritizing high mean squares before low mean squares, considering positive ZSTD before negative ZSTD, and starting with the worst item or person. After excluding the “worst” item or person, there will always be another item or person that may appear as the “worst” in the newly adjusted context, which is more suitable for the model. Therefore, it is important not to mechanically remove items as this may result in no remaining items or persons. In other words, the ideal range for fitting suitable individuals should fall within the required values [66,67]. Those individuals who have statistically exceeded the acceptable threshold, either by correctly answering items that are more difficult than their abilities or by failing to answer correctly to items that require lower abilities than their own, might have relied on guessing, lacked in seriousness, or provided inaccurate responses [66,67]. By employing these data analysis techniques, the researcher aimed to validate the factor structure and unidimensionality of the Perceived Collective Family Efficacy Scale in the Arab context. These rigorous analyses contribute to evaluating the scale’s psychometric properties and establish its suitability for assessing perceived collective family efficacy among Arab populations. The statistical analyses were performed using AMOS 24.0 (for CFA), Winsteps (for Rasch), and SPSS 24.0 (for other analyses).

## 3. Results

### 3.1. Sociodemographic Characteristics of the Sample

The sociodemographic characteristics of the sample are summarized in Table 1. The number of females was 255 (85%). Regarding the number of siblings, 49% of participants had one to four siblings, followed by 43% having five to eight siblings, and 8% having more than nine siblings. Notably, the majority of the sample were single individuals, with a percentage of 92.7%, while the percentage of married individuals was low, estimated at 7.3%. A significant proportion of the sample reported that their parents lived together, accounting for 84.3%, while the percentage of individuals with divorced parents was 3.7%, and 12% had one or both parents deceased. The economic level of the majority of the sample’s families was moderate, with a percentage of 85.3%, followed by a low economic level of 6%, and a small percentage of 8.7% had a high economic level. The most common field of study among the students was humanities and social sciences, accounting for 84.7%, followed by natural sciences with a percentage of 9.3%. Finally, the percentage of students in the arts and languages field was 6%.

### 3.2. Descriptive Statistics

The descriptive statistics for the scale items are presented in Table 2, which includes the skewness, kurtosis, mean, and standard deviation (SD) for each item individually. These statistics summarize the central tendencies and variability of each item in the scale.

The skewness values ranged from −0.915 to −0.05, indicating a normal distribution of the items. The kurtosis values ranged from −0.741 to 0.371, also indicating a normal distribution. The mean values ranged from 3.18 to 4.01. The item “Serve as a positive example for the community” had the highest mean, while the rest of the items had means above 3.

### 3.3. Confirmatory Factor Analysis

**Figure 1:**
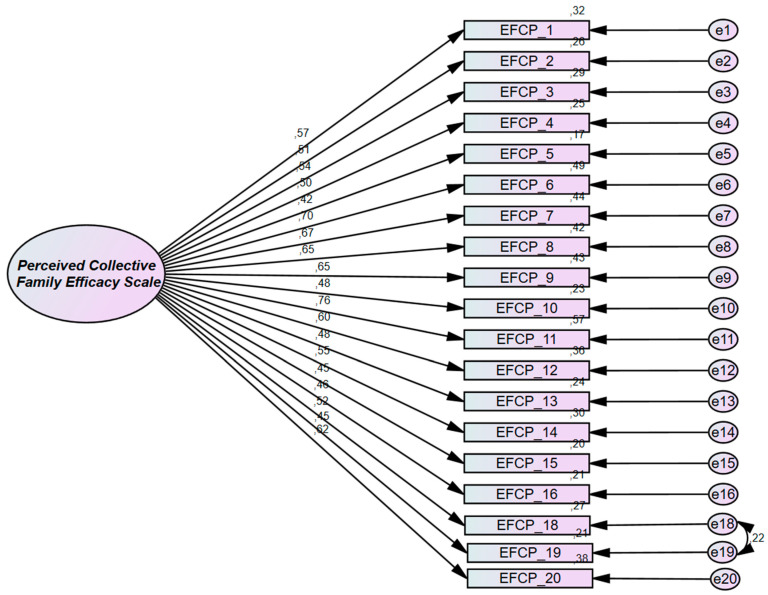
Perceived Collective Family Efficacy Scale in its final form after modification through confirmatory factor analysis.

Table 3 presents the CFA fit indices. Overall, the results of the fit indices indicate a good model fit after modification. The initial model fit was unsatisfactory, such as the CFI value at 0.878. After making two modifications (i.e., deleting item 17 and linking up the residual correlation between items 18 and 19), the fit indices were acceptable: CFI = 0.912, SRMR = 0.04, and RMSEA = 0.05. Regarding the loadings of the items after conducting CFA, as shown in Figure 1, they ranged from 0.418 (item 5) to 0.756 (item 11), all of which are acceptable loadings.

#### Model Validity

The model’s Cronbach’s alpha value was 0.898, indicating high internal consistency, and the Composite Reliability value was 0.896. These values are considered good, suggesting a strong reliability of the model. Furthermore, the MaxR(H) value of 0.907 exceeded the CR value, which indicates the establishment of discriminant validity. This implies that the constructs in the model measure different aspects of the Perceived Collective Family Efficacy under investigation. Overall, these findings provide further support for the validity and reliability of the model.

### 3.4. Rasch Analysis

#### 3.4.1. Fit of Individuals and Items to Rasch Analysis

The participants’ Infit mean-square (IN.MSQ) values ranged from 0.1132 to 2.8103, and Outfit mean square ranged from 0.1141 to 2.8711, indicating a general fit of persons to the Rasch model. Regarding the fit of items to the Rasch analysis (see Table 4), the Outfit MnSq ranged from 0.79 to 1.38, while the Infit MnSq ranged from 0.81 to 1.33. These values align well with the Rasch model, as the items do not exceed the fit boundaries of 0.60 and 1.40.

**Figure 2:**
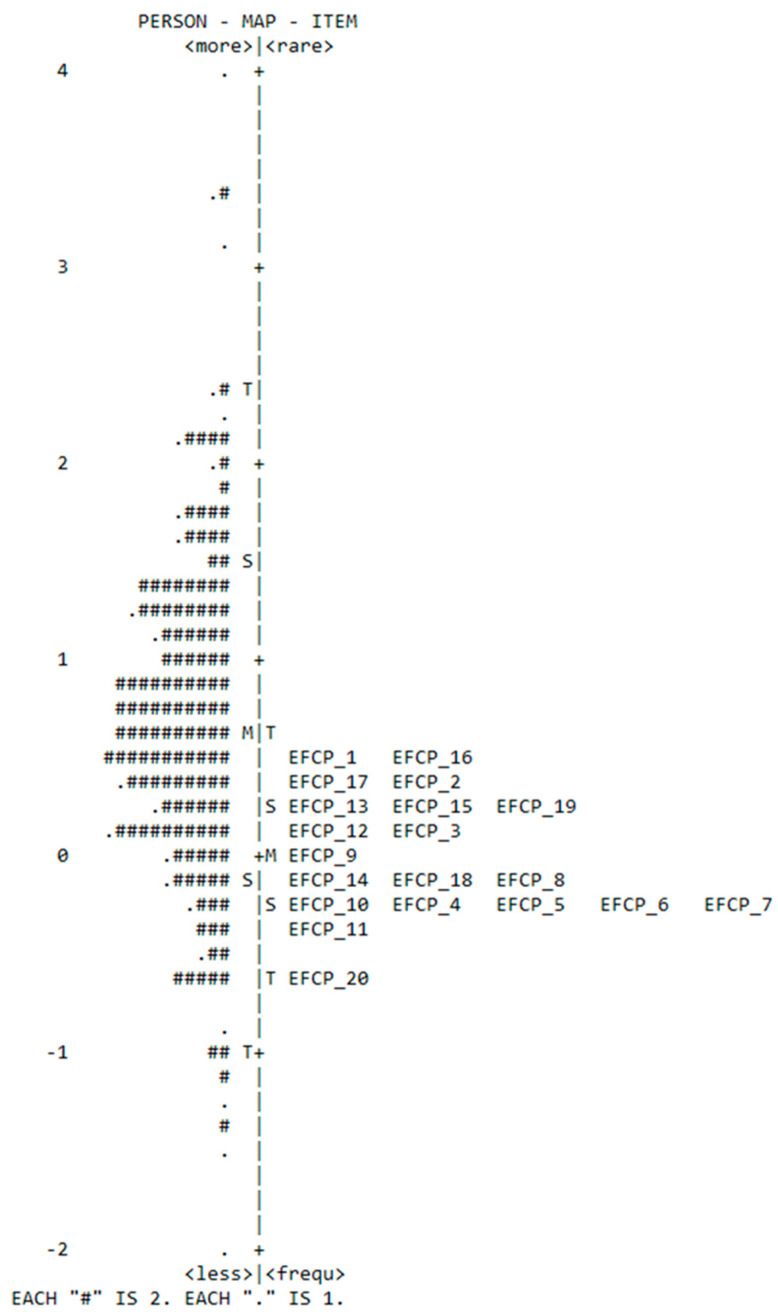
Grading the difficulty of items and assessing individuals’ abilities based on the distribution map.

In this study, the item difficulty values ranged from −0.66 to 0.48, as shown in the table above. The logit value (0) was not observed in items with moderate difficulty, indicating the absence of items with moderate difficulty. However, the logit values were positive for items with higher-than-moderate difficulty and deviated from zero. Specifically, the items with positive logits were 12, 3, 19, 15, 13, 2, 17, 16, and 1. On the other hand, items with lower difficulty had negative logits, as represented by the following items: 20, 11, 7, 6, 4, 10, 5, 8, 14, 18, and 9 (see Figure 2). The average logit difficulty score was 0, with a standard deviation of 0.30. The average score and standard deviation in item difficulty logit suggest homogeneity and proximity to the mean (0) logit, indicating item consistency and uniformity.

Through the grading map in Figure 2, we observe that it illustrates the order of items, ranging from (−1 to +1). Furthermore, the map reveals the presence of the ceiling effect, which means that individuals with high abilities do not encounter items that challenge their proficiency beyond a certain level. However, the map does not measure high proficiency accurately (meaning that we need items that match the abilities of individuals with high capabilities).

#### 3.4.2. Empirical Item Characteristic Curves (ICCs)

**Figure 3:**
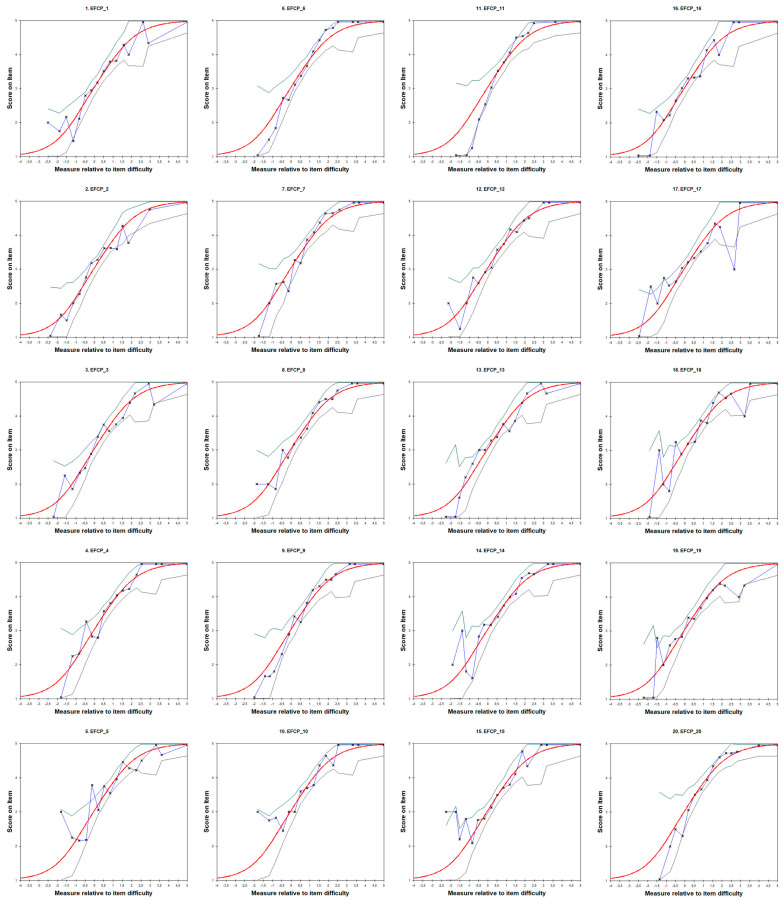
Empirical Item Characteristic Curves (ICCs) of the Perceived Collective Family Efficacy Scale. Note: The red line represents the item characteristic curve, as predicted by the Rasch model. It shows the average score that students at different levels of the latent variable (*x*-axis) would obtain on the item (*y*-axis) according to the Rasch model. The blue line depicts the empirical ICC. Each “x” on the *x*-axis summarizes the responses of students whose measurements are near that particular point. The green-gray lines represent the two-sided 95% confidence bands.

We checked the fit of the items using Empirical Item Characteristic Curves (ICCs). We found that all items fit within the two-sided 95% confidence bands, except for item 17, which showed a misfit. Figure 3 shows that item 17’s empirical data fell outside the confidence bands.

#### 3.4.3. Unidimensionality of the Perceived Collective Family Efficacy Scale

Due to the assumption of unidimensionality in the Rasch model, it should be noted that unidimensionality is not absolute. Unidimensionality should not be equated with factor analysis, as their goals differ. Factor analysis aims to identify the factors that make up the test, while item response theory aims to identify deviations from the measured trait and determine whether they constitute an independent factor. Therefore, the software provides Rasch residual-based Principal Component Analysis (PCAR) to analyze the underlying dimensions, as shown in Table 5. This analysis reveals differences between dimensions and allows for an assessment of unidimensionality based on the following criteria:

(a) The variance explained by measures should be greater than or equal to 20% to 80% (in our study, the variance explained was 36.3%, which is good).
(b) The raw variance explained by items (36.3%) is larger than the raw variance explained by persons (14.4%).
(c) At most, five contrasts are reported, and in our model, there are five variances.
(d) All conditions for the unidimensionality of Rasch are acceptable, as shown in the table above, except for the unexplained variance in the first contrast, which is 2.1, slightly higher than the recommended 2.0.

#### 3.4.4. Reliability

The Rasch model provides an overall test reliability coefficient, as in classical measurement theory, and reliability coefficients for items and persons. It is evident that the item separation coefficient for the test was estimated at 4.23, which exceeds 2. This confirms the hierarchical ordering of the scale items based on item difficulty. The item reliability value was 0.95, indicating high reliability. The person separation coefficient was 2.62, which is greater than 2. Moreover, the person reliability was 0.87, a good value indicating scale stability. This suggests that individuals can effectively differentiate between the items, accurately defining the targeted trait.

#### 3.4.5. Response Category Functioning of the Perceived Collective Family Efficacy Scale

**Figure 4:**
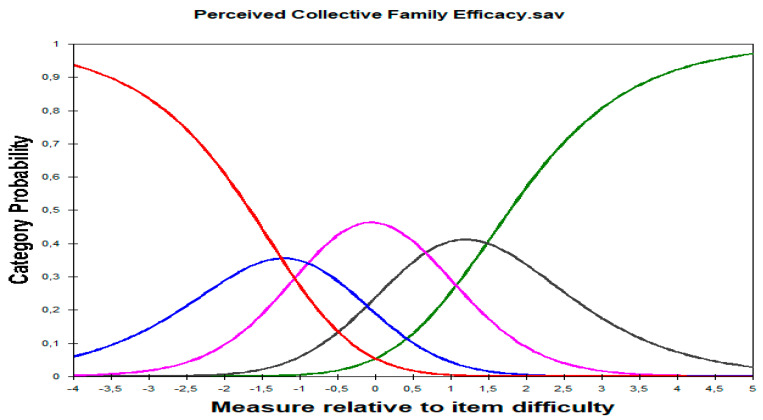
Measure relative to item difficulty: five categories. Note: The horizontal axis shows the measured variable, while the vertical axis displays the probability of choosing a category between 1 and 5. Each curve represents responses on a five-point Likert scale, with ’Never’ represented in red, ’Rarely’ in blue, ’Sometimes’ in pink, ’Often’ in gray, and ’Very often’ in green.

The analysis of category performance under the Rasch measurement requirements is presented in Table 6 and illustrated in Figure 4. It shows the category probability curves for the Perceived Collective Family Efficacy Scale, ranging from 1 (not at all well) to 5 (very well). As shown in Figure 4, the graphs demonstrate the likelihood of individuals selecting various categories for Perceived Collective Family Efficacy. The horizontal axis shows the measured variable, while the vertical axis displays the probability of choosing a category between 1 and 5. Each curve represents responses on a five-point Likert scale, with ‘Never’ represented in red, ‘Rarely’ in blue, ‘Sometimes’ in pink, ‘Often’ in gray, and ‘Very often’ in green.

The perfect graph would exhibit a peak for each category. An analysis was conducted to verify the effectiveness of the five-category Likert response format. The results demonstrated a consistent distribution of responses, logit measures increased as categories increased, and outfit statistics within the range (<2.0). The sequential arrangement of category thresholds suggested that the 5-category rating scale performed optimally.

Both Infit and Outfit MnSq ranged from 0.6 to 1.4, which are considered acceptable for rating scale measurement [66]. Table 6 confirms that none of the values exceeded 1.40 or fell below 0.60. The observed average person measures for respondents endorsing each category progressed monotonically with the categories: −0.54 < −0.08 < 0.38 < 0.87 < 1.40. This pattern indicates that individuals with higher abilities endorse higher categories, while those with lower abilities support lower categories [66]. Regarding the thresholds between categories, it is optimal for the Andrich threshold step values to have a minimum difference in step difficulty of 1.4 logits for an optimum response category performance. From Table 6, it is found that the width between the Andrich thresholds for categories 1 and 2 is −1.25 logits, categories 2 and 3 is (−1.25) + (−0.89) = 2.14 logits, categories 3 and 4 is 1.55 logits, and categories 4 and 5 is 2.13 logits.

## 4. Discussion

The study’s results demonstrated that the proposed single-factor structure for the Perceived Collective Family Efficacy Scale exhibited an acceptable fit in both CFA and Rasch results. The CFA results supported the single-factor structural model, and it was found that the scale possesses good validity and reliability after being analyzed through the Rasch model. This finding is consistent with previous studies [24,27,28,29]. The study extended the psychometric properties of the Perceived Collective Family Efficacy Scale to the Arab context (i.e., in Algeria). The findings revealed that the scale exhibited acceptable indicators of quality, supported by confirmatory factor analysis. This suggests that the scale effectively measures the Algerian population’s perceived collective family efficacy construct. The single-factor structural model was validated, confirming the theoretical framework of the scale. These results align with previous studies [24,27,28] conducted in different cultural contexts, indicating the generalizability of the scale’s psychometric properties. Our study in Algeria has echoed well with recent findings reported in Italian and Portuguese participants [27]. Other prior results suggest that the Perceived Collective Family Efficacy Scale is applicable across different cultures: Caprara’s study in 2004 supported the scale’s unidimensionality and good reliability among family participants from Genzano, a residential community near Rome, and from Milan and its surroundings [24], Pepe et al.’s study in 2008 also supported the scale’s unidimensionality and good reliability among Spanish participants [28]. Our study findings extended the psychometric evidence of the Perceived Collective Family Efficacy Scale to Algeria with a reliability of 0.898. Overall, our study enhances the scale’s applicability in Arab cultures, with Algeria serving as a model for its validity and unidimensional structure.

The scale demonstrated good levels of validity and reliability in the Algerian context, implying that it could accurately measure the intended construct and produce results consistent with prior findings [24]. This finding further enhances the scale’s utility and applicability in an Arab context. The study’s findings contribute to the existing body of literature on perceived collective family efficacy and provide valuable insights into its measurement and psychometric properties. Researchers and practitioners can confidently employ the Perceived Collective Family Efficacy Scale in the Algerian context to assess and understand the collective efficacy beliefs within families. The scale’s validity and reliability establish a foundation for future research and interventions to promote family efficacy and well-being in similar cultural settings. The findings support its applicability in an Arab cultural context and contribute to the broader understanding of family dynamics and functioning. Future research can build upon these findings by exploring the scale’s associations with other relevant variables and examining its effectiveness in intervention programs to strengthen family efficacy and resilience.

### 4.1. Implications

#### 4.1.1. Theoretical Implications

The study establishes the validity and reliability of the Perceived Collective Family Efficacy Scale in the Algerian context. This provides a foundation for future research and ensures the credibility of findings based on the scale. That is, the scale can be used in diverse populations and cultural settings, enhancing its utility as a cross-cultural measurement tool. The validation of the single-factor structural model and alignment with theoretical assumptions (unidimensionality, reliability, validity) support the conceptual understanding of perceived collective family efficacy. The study highlights the applicability of the Perceived Collective Family Efficacy Scale in an Arab cultural context, contributing to the broader understanding of family dynamics and functioning within Arab societies.

#### 4.1.2. Practical Implications

The study findings could assist healthcare practitioners in assessing and understanding collective efficacy beliefs within families. Additionally, the findings hold practical implications for healthcare practitioners involved in designing intervention programs aimed at promoting family efficacy and well-being within the Algerian context. The scale’s established validity and reliability ensure its effective utilization for evaluating the impact of interventions on enhancing family efficacy. Practitioners can thus customize their interventions based on the scale’s measurements and pinpoint areas for improvement within families. This underscores the importance of considering cultural factors when assessing and promoting family efficacy, highlighting the necessity for culturally sensitive interventions. The Perceived Collective Family Efficacy Scale is culturally appropriate for use with other Arabic-speaking populations. The scale’s formulation and items align with the cultural values of various Arab countries. This suitability was demonstrated through the scale’s validity and reliability within the Algerian Arab population in the present study. For instance, Cronbach’s alpha coefficient for the collective family scale demonstrated a high internal consistency.

## 5. Limitations and Recommendations

### 5.1. Limitations

There are a few limitations to this study that should be addressed. Firstly, it is important to note that the sample used for this study was drawn from only one university, so caution should be taken when trying to generalize the results to other student populations. Secondly, this study focused only on a sample of students with a low representation of males. Previous studies have shown that gender differences in Algeria’s education system are clear [68,69], with girls consistently performing better than boys in secondary school and university. This gender gap has widened over the years, with women making up nearly 60% of university students. It is worth noting that women tend to dominate areas such as education, humanities, social sciences, and health and welfare, while men display a stronger inclination toward pursuing STEM (science, technology, engineering, and mathematics) disciplines. Lastly, the study’s reliance solely on confirmatory factor analysis and the Rasch model for assessing validity may represent a limitation. Using additional instruments to evaluate the study’s concurrent, convergent, and divergent validity would be beneficial.

### 5.2. Recommendations for Future Research

In order to improve the generalizability of the study’s findings, we propose that similar research be conducted in other Algerian and Arab universities from different specializations with random sampling, as they speak the same language. It would be valuable to explore the psychometrics of this scale across a variety of sample sizes and age groups, including adolescents and adults with equal gender representation. Additionally, we suggest using different statistical methods such as retesting, Exploratory Factor Analysis (EFA), and Differential Item Functioning (DIF) analysis to evaluate the scale’s validity and reliability comprehensively.

To improve future research in this field, it is recommended to use multiple measurement tools to ensure data accuracy. To establish concurrent validity, we recommend comparing the results with established measures of the same concept, as well as examining relationships with related and unrelated concepts to assess convergent and divergent validity. Test–retest assessments should also be conducted to ensure measurement reliability over time. These methodological improvements will strengthen the validity of the study’s findings and contribute to a comprehensive validation process.

## 6. Conclusions

In conclusion, this study examined the psychometric properties of the Arabic version of the Perceived Collective Family Efficacy Scale by employing CFA and the Rasch model with a sample of university students. The study’s findings demonstrate that the Perceived Collective Family Efficacy Scale exhibits satisfactory validity and reliability within the Algerian context. The established validity and reliability of the scale provide a foundation for future investigations and interventions aimed at promoting family efficacy and well-being in similar cultural contexts. Further research can explore its associations with other variables and assess its effectiveness in family efficacy and resilience intervention programs.

## Figures and Tables

**Table 1:** Characteristics of the study sample.

Variables	Groups	N	%
Gender	Boys	45	15.0
	Girls	255	85.0
Number of siblings	1–4	147	49.0
	5–8	129	43.0
	>9	24	8.0
Marital status	Single	278	92.7
	Married	22	7.3
Parental status	Live together	253	84.3
	Divorced	11	3.7
	One or both of them is dead	36	12.0
Family economic status	Lower	18	6.0
	Middle	256	85.3
	Upper	26	8.7
Specialties	Social and human sciences	254	84.7
	Natural sciences	28	9.3
	Literature and language	18	6.0

**Table 2:** Descriptive statistics for The Perceived Collective Family Efficacy Scale.

Items	Skewness	Kurtosis	Mean	SD
Set aside leisure time with your family when other things press for attention.	−0.189	−0.300	3.18	1.06
Agree to decisions that require some sacrifice of personal interests.	−0.208	−0.326	3.28	1.01
Resolve conflicts when family members feel they are not being treated fairly.	−0.308	−0.174	3.42	0.99
Prevent family disagreements from turning into heated arguments.	−0.560	−0.181	3.71	1.06
Get family members to share household responsibilities.	−0.604	−0.278	3.71	1.10
Support each other in times of stress.	−0.531	−0.464	3.71	1.09
Help each other to achieve their personal goals.	−0.617	−0.223	3.75	1.07
Help each other with work demands.	−0.362	−0.448	3.68	1.00
Build respect for each other’s particular interests.	−0.481	−0.395	3.58	1.09
Get family members to carry out their responsibilities when they neglect them.	−0.550	−0.331	3.71	1.07
Build trust in each other.	−0.764	0.124	3.78	1.08
Figure out what choices to make when the family faces important decisions.	−0.403	−0.235	3.48	1.05
Find community resources and make good use of them for the family.	−0.312	−0.193	3.37	1.04
Get the family to keep close ties to their larger family.	−0.469	−0.151	3.64	1.01
Celebrate family traditions even in difficult times.	−0.144	−0.677	3.38	1.12
Cooperate with schools to improve their educational practices.	−0.094	−0.741	3.18	1.17
Face up to difficulties without excessive tension.	−0.050	−0.349	3.22	0.97
Remain confident during difficult times.	−0.398	−0.548	3.65	1.06
Accept each member’s need for independence.	−0.261	−0.364	3.40	1.02
Serve as a positive example for the community.	−0.915	0.371	4.01	1.01

**Table 3:** Model fit.

Model Fit	Without Modifications	With Modifications
χ^2^	390.211	299.780
χ^2^/df	2.295	1.985
CFI	0.878	0.912
SRMR	0.05	0.04
RMSEA	0.06	0.05

**Table 4:** Item statistics: Measure order.

Items	Measure	Error	IN.MSQ	IN.ZSTD	OUT.MSQ	OUT.ZSTD
EFCP_1	0.48	0.06	0.9079	−1.1991	0.9471	−0.6691
EFCP_2	0.35	0.07	0.9252	−0.9591	0.9585	−0.519
EFCP_3	0.17	0.07	0.8721	−1.6891	0.9195	−1.0291
EFCP_4	−0.22	0.07	1.109	1.3711	1.1048	1.2911
EFCP_5	−0.22	0.07	1.3305	3.8513	1.3847	4.3314
EFCP_6	−0.22	0.07	0.9137	−1.1091	0.8859	−1.4591
EFCP_7	−0.28	0.07	0.9627	−0.449	0.9328	−0.8291
EFCP_8	−0.18	0.07	0.8118	−2.5392	0.7902	−2.8192
EFCP_9	−0.05	0.07	0.9331	−0.8491	0.9118	−1.1291
EFCP_10	−0.22	0.07	1.1817	2.2112	1.1312	1.6011
EFCP_11	−0.31	0.07	0.8222	−2.3792	0.79	−2.7792
EFCP_12	0.09	0.07	0.9259	−0.9491	0.9064	−1.2091
EFCP_13	0.24	0.07	1.054	0.7111	1.0503	0.6611
EFCP_14	−0.13	0.07	0.9164	−1.0691	0.8909	−1.3991
EFCP_15	0.22	0.07	1.2206	2.6812	1.2222	2.6812
EFCP_16	0.47	0.06	1.2196	2.6812	1.1984	2.4312
EFCP_17	0.43	0.06	0.923	−0.9991	0.9421	−0.7391
EFCP_18	−0.13	0.07	1.0245	0.341	1.0081	0.131
EFCP_19	0.19	0.07	0.9865	−0.149	1.0368	0.491
EFCP_20	−0.66	0.07	1.0108	0.161	0.945	−0.6291

**Table 5:** Unidimensionality of the Perceived Collective Family Efficacy Scale.

	Empirical		Modeled
Total raw variance in observations	31.4	100.0%	100.0%
Raw variance explained by measures	11.4	36.3%	36.5%
Raw variance explained by persons	4.5	14.4%	14.5%
Raw variance explained by items	6.9	21.8%	22.0%
Raw unexplained variance (total)	20.0	63.7%	63.5%
Unexplained variance in 1st contrast	2.1	6.6%	10.4%
Unexplained variance in 2nd contrast	1.7	5.4%	8.4%
Unexplained variance in 3rd contrast	1.5	4.8%	7.5%
Unexplained variance in 4th contrast	1.4	4.3%	6.8%
Unexplained variance in 5th contrast	1.3	4.2%	6.6%

**Table 6:** Summary of category structure. Model = “R”.

Category		Observed		Observed	Sample	Infit	Outfit	Structure	Category
Label	Score	Count	%	Average	Expect	Mnsq	Mnsq	Calibration	Measure
1	1	265	4	−0.54	−0.61	1.08	1.11	NONE	(−2.69)
2	2	662	11	−0.08	−0.07	0.99	0.98	−1.25	−1.21
3	3	1914	32	0.38	0.40	0.94	0.93	−0.89	−0.06
4	4	1849	31	0.87	0.86	0.94	0.92	0.66	1.19
5	5	1310	22	1.40	1.39	1.04	1.03	1.47	(2.82)

## Data Availability

The dataset supporting this study’s findings is not openly available and will be available from the corresponding author upon reasonable request.

## Supplementary Materials

The following supporting information can be downloaded at: https://www.mdpi.com/article/10.3390/healthcare11192691/s1, Table S1: English version of The Perceived Collective Family Efficacy Scale; Table S2: Arabic version of The Perceived Collective Family Efficacy Scale; Table S3: Back-translation version of The Perceived Collective Family Efficacy Scale.

## References

[1] Gilbertson S., Graves B.A., Watson R.R., Zibadi S. (2018). Chapter 4—Heart Health and Children. Lifestyle in Heart Health and Disease.

[2] McGinnis H.A., Wright A.W., Halpern-Felsher B. (2023). Adoption and child health and psychosocial well-being. Encyclopedia of Child and Adolescent Health.

[3] Aiche S. (2021). Building a measure of functional family performance: A field study on a sample of respondents in Algeria. J. Educ. Qual. Res..

[4] Taresh S.M., Ahmad N.A., Roslan S., Ma’rof A.M. (2020). Preschool Teachers’ Beliefs towards Children with Autism Spectrum Disorder (ASD) in Yemen. Children.

[5] Schermerhorn A.C., Mark Cummings E., Kail R.V. (2008). Transactional Family Dynamics: A New Framework for Conceptualizing Family Influence Processes. Advances in Child Development and Behavior.

[6] Watson W.H., Ramachandran V.S. (2012). Family Systems. Encyclopedia of Human Behavior.

[7] Aiche S., Hammadi F. (2023). The factorial structure of the satisfaction with family life scale on a sample of Algerians. Al-Qabas J. Psychol. Soc. Stud..

[8] Caporino N.E., Peris T.S., Storch E.A., McGuire J.F. (2020). Chapter 14—Involving family members in exposure therapy for children and adolescents. Exposure Therapy for Children with Anxiety and OCD.

[9] Coulacoglou C., Saklofske D.H., Coulacoglou C., Saklofske D.H. (2017). Chapter 8—The Assessment of Family, Parenting, and Child Outcomes. Psychometrics and Psychological Assessment.

[10] Conner M., Wright J.D. (2015). Health Behaviors. International Encyclopedia of the Social & Behavioral Sciences.

[11] Johnson D.W., Johnson R.T., Wright J.D. (2015). Cooperation and Competition. International Encyclopedia of the Social & Behavioral Sciences.

[12] Schunk D.H., DiBenedetto M.K., Tierney R.J., Rizvi F., Ercikan K. (2023). Learning from a social cognitive theory perspective. International Encyclopedia of Education.

[13] Procentese F., Gatti F., Di Napoli I. (2019). Families and Social Media Use: The Role of Parents’ Perceptions about Social Media Impact on Family Systems in the Relationship between Family Collective Efficacy and Open Communication. Int. J. Environ. Res. Public Health.

[14] Bandura A. (2001). Social cognitive theory: An agentic perspective. Annu. Rev. Psychol..

[15] Urdan T., Pajares F. (2006). Self-Efficacy Beliefs of Adolescents.

[16] Butler J., Gellman M.D. (2020). Self-Efficacy. Encyclopedia of Behavioral Medicine.

[17] Bandura A. (1997). Self-Efficacy: The Exercise of Control.

[18] Donohoo J., Hattie J., Eells R. (2018). The power of collective efficacy. Educ. Leadersh..

[19] Pietrantoni L., Michalos A.C. (2014). Collective Efficacy. Encyclopedia of Quality of Life and Well-Being Research.

[20] Dehingia N., Dixit A., Heskett K., Raj A. (2022). Collective efficacy measures for women and girls in low- and middle-income countries: A systematic review. BMC Women’s Health.

[21] Aiche S. (2021). Family Sacrifice: A Theoretical Approach. Soc. Empower. J..

[22] Bandura A., Caprara G.V., Barbaranelli C., Regalia C., Scabini E. (2011). Impact of family efficacy beliefs on quality of family functioning and satisfaction with family life. Appl. Psychol..

[23] Scabini E., Marta E., Lanz M. (2007). The Transition to Adulthood and Family Relations: An Intergenerational Approach.

[24] Caprara G.V., Regalia C., Scabini E., Barbaranelli C., Bandura A. (2004). Assessment of Filial, Parental, Marital, and Collective Family Efficacy Beliefs. Eur. J. Psychol. Assess..

[25] Kao T.-S.A., Ling J., Dalaly M., Robbins L.B., Cui Y. (2020). Parent–Child Dyad’s Collective Family Efficacy and Risky Adolescent Health Behaviors. Nurs. Res..

[26] Caprara G.V. (2001). La Valutazione Dell’autoefficacia. Costrutti e Strumenti.

[27] Costa M., Faria L., Alessandri G., Caprara G.V. (2016). Measuring parental and family efficacy beliefs of adolescents’ parents: Cross-cultural comparisons in Italy and Portugal. Int. J. Psychol..

[28] Pepe S., Sobral J., Gómez-Fraguela J.A., Villar-Torres P. (2008). Spanish adaptation of the Adolescents’ perceived collective family efficacy scale. Psicothema.

[29] Panaghi L., Mokhtarnai I., Kalantary F. (2016). A Preliminary Study of Psychometric Properties of the Adolescents’ Perceived Family Collective Efficacy Scale in Adolescent. J. Fam. Res..

[30] Sabah A., Khalaf Rashid Al-Shujairi O., Boumediene S. (2021). The Arabic Version of the Walsh Family Resilience Questionnaire: Confirmatory Factor Analysis of a Family Resilience Assessment Among Algerian and Iraq Families. Int. J. Syst. Ther..

[31] Aljaberi M.A., Al-Sharafi M.A., Uzir M.U.H., Sabah A., Ali A.M., Lee K.-H., Alsalahi A., Noman S., Lin C.-Y. (2023). Psychological Toll of the COVID-19 Pandemic: An In-Depth Exploration of Anxiety, Depression, and Insomnia and the Influence of Quarantine Measures on Daily Life. Healthcare.

[32] Sabah A., Boumediene S., Zineb D. (2021). Adverse Life Events and Family Distress During the Coronavirus Pandemic: A Field Study in Algeria. Arab. J. Psychiatry.

[33] Aljaberi M.A., Alareqe N.A., Alsalahi A., Qasem M.A., Noman S., Uzir M.U.H., Mohammed L.A., Fares Z.E.A., Lin C.-Y., Abdallah A.M. (2022). A cross-sectional study on the impact of the COVID-19 pandemic on psychological outcomes: Multiple indicators and multiple causes modeling. PLoS ONE.

[34] Hunziker S., Blankenagel M. (2021). Cross-Sectional Research Design. Research Design in Business and Management: A Practical Guide for Students and Researchers.

[35] Daniels C.D., Goldstein S., Naglieri J.A. (2011). Cross-Sectional Research. Encyclopedia of Child Behavior and Development.

[36] Voelkle M.C., Hecht M., Zeigler-Hill V., Shackelford T.K. (2017). Cross-Sectional Research Designs. Encyclopedia of Personality and Individual Differences.

[37] Hair J., Black W., Babin B., Anderson R. (2019). Multivariate Data Analysis.

[38] Kline R.B. (2023). Principles and Practice of Structural Equation Modeling.

[39] Alareqe N.A., Hassan S.A., Kamarudin E.M.E., Aljaberi M.A., Nordin M.S., Ashureay N.M., Mohammed L.A. (2022). Validity of Adult Psychopathology Model Using Psychiatric Patient Sample from a Developing Country: Confirmatory Factor Analysis. Ment. Illn..

[40] Kline R.B. (2016). Principles and Practice of Structural Equation Modeling.

[41] Selig J.P., Card N.A., Little T.D. (2014). Latent variable structural equation modeling in cross-cultural research: Multigroup and multilevel approaches. Multilevel Analysis of Individuals and Cultures.

[42] Jackson D.L. (2007). The effect of the number of observations per parameter in misspecified confirmatory factor analytic models. Struct. Equ. Model. A Multidiscip. J..

[43] Jackson D.L. (2003). Revisiting sample size and number of parameter estimates: Some support for the N:q hypothesis. Struct. Equ. Model..

[44] Keith T.Z. (2014). Multiple Regression and Beyond: An Introduction to Multiple Regression and Structural Equation Modeling.

[45] Nichols A.L., Edlund J. (2023). The Cambridge Handbook of Research Methods and Statistics for the Social and Behavioral Sciences: Volume 1: Building a Program of Research.

[46] Chapman D.W., Carter J.F. (1979). Translation Procedures for the Cross Cultural Use of Measurement Instruments. Educ. Eval. Policy Anal..

[47] Brislin R.W. (1986). The wording and translation of research instruments. Field Methods in Cross-Cultural Research.

[48] Bravo M., Canino G.J., Rubio-Stipec M., Woodbury-Fariña M. (1991). A cross-cultural adaptation of a psychiatric epidemiologic instrument: The diagnostic interview schedule’s adaptation in Puerto Rico. Cult. Med. Psychiatry.

[49] Zaid S.M.R., Jamaluddin S., Baharuldin Z., Taresh S.M. (2020). Psychometric properties of an adapted Yemeni version of rejection sensitivity questionnaire. Pertanika J. Soc. Sci. Humanit..

[50] Kelley K., Clark B., Brown V., Sitzia J. (2003). Good practice in the conduct and reporting of survey research. Int. J. Qual. Health Care.

[51] Nadhiroh S., Nurmala I., Pramukti I., Tivany S., Tyas L., Zari A., Poon W., Siaw Y.-L., Kamolthip R., Chirawat P. (2022). Weight stigma in Indonesian young adults: Validating the indonesian versions of the weight self-stigma questionnaire and perceived weight stigma scale. Asian J. Soc. Health Behav..

[52] World Health Organization Process of Translation and Adaptation of Instruments. http://www.who.int/substance_abuse/research_tools/translation/en/.

[53] Hair J.F., Hult G.T.M., Ringle C.M., Sarstedt M. (2021). A Primer on Partial Least Squares Structural Equation Modeling (PLS-SEM).

[54] Aljaberi M.A., Juni M.H., Al-Maqtari R.A., Lye M.S., Saeed M.A., Al-Dubai S.A.R., Kadir Shahar H. (2018). Relationships among perceived quality of healthcare services, satisfaction and behavioural intentions of international students in Kuala Lumpur, Malaysia: A cross-sectional study. BMJ Open.

[55] Mohammed L.A., Aljaberi M.A., Amidi A., Abdulsalam R., Lin C.-Y., Hamat R.A., Abdallah A.M. (2022). Exploring Factors Affecting Graduate Students&rsquo; Satisfaction toward E-Learning in the Era of the COVID-19 Crisis. Eur. J. Investig. Health Psychol. Educ..

[56] Aljaberi M.A., Lee K.-H., Alareqe N.A., Qasem M.A., Alsalahi A., Abdallah A.M., Noman S., Al-Tammemi A.A.B., Mohamed Ibrahim M.I., Lin C.-Y. (2022). Rasch Modeling and Multilevel Confirmatory Factor Analysis for the Usability of the Impact of Event Scale-Revised (IES-R) during the COVID-19 Pandemic. Healthcare.

[57] Abiddine F.Z.E., Aljaberi M.A., Gadelrab H.F., Lin C.-Y., Muhammed A. (2022). Mediated effects of insomnia in the association between problematic social media use and subjective well-being among university students during COVID-19 pandemic. Sleep Epidemiol..

[58] Alareqe N.A., Roslan S., Taresh S.M., Nordin M.S. (2021). Universality and Normativity of the Attachment Theory in Non-Western Psychiatric and Non-Psychiatric Samples: Multiple Group Confirmatory Factor Analysis (CFA). Int. J. Environ. Res. Public Health.

[59] Fekih L. (2018). Bullying among high school students and their relationship with diligence at school Field study on a sample of secondary school students in Algeria. Qatar Found. Annu. Res. Conf. Proc..

[60] Sabah A., Aljaberi M.A., Lin C.-Y., Chen H.-P. (2022). The Associations between Sibling Victimization, Sibling Bullying, Parental Acceptance&ndash;Rejection, and School Bullying. Int. J. Environ. Res. Public Health.

[61] Alareqe N.A., Roslan S., Nordin M.S., Ahmad N.A., Taresh S.M. (2021). Psychometric Properties of the Millon Clinical Multiaxial Inventory–III in an Arabic Clinical Sample Compared With American, Italian, and Dutch Cultures. Front. Psychol..

[62] Sabah A., Al-Shujairi O.K.R. (2022). The Confirmatory Factor Analysis of the Spiritual Wellbeing Scale of a Sample of Students from Iraq and Algeria. J. STEPS Humanit. Soc. Sci..

[63] Kimberlin C.L., Winterstein A.G. (2008). Validity and reliability of measurement instruments used in research. Am. J. Health-Syst. Pharm..

[64] Bond T.G., Fox C.M. (2013). Applying the Rasch Model: Fundamental Measurement in the Human Sciences.

[65] Linacre J.M. (2006). WINSTEPS Rasch Measurement Computer Program.

[66] Bond T.G., Fox C.M. (2015). Applying the Rasch Model: Fundamental Measurement in the Human Sciences.

[67] Ariffin S.R., Omar B., Isa A., Sharif S. (2010). Validity and reliability multiple intelligent item using rasch measurement model. Procedia-Soc. Behav. Sci..

[68] Zahia O.-B. (2018). Gender inequity in education in Algeria: When inequalities are reversed. J. Educ. Soc. Policy.

[69] AHMAID I. (2021). What Stands behind the Balanced Ratio of Male/Female Students in the Algerian STEM Education Despite the Country’s Low Gender Equity?. Master’s Thesis.

