# Supplementary material for: Psychometric Properties of the Perceived Collective Family Efficacy Scale in Algeria
Source: Healthcare (Basel). 2023 Oct 8;11(19):2691. doi: 10.3390/healthcare11192691 (PMC10572840; doi:10.3390/healthcare11192691)
Supplement: Supplementary file 1 [file healthcare-11-02691-s001.zip › healthcare-2577692-supplementary.pdf]

Supplementary Table S1. English version of The Perceived Collective Family Efficacy scale

No	Items	Not at all (1)	Poorly (2)	Moderately (3)	Well (4)	Very well (5)
1	Set aside leisure time with your family when other things press for attention.					
2	Agree to decisions that require some sacrifice of personal interests.					
3	Resolve conflicts when family members feel they are not being treated fairly.					
4	Prevent family disagreements from turning into heated arguments.					
5	Get family members to share household responsibilities.					
6	Support each other in times of stress.					
7	Help each other to achieve their personal goals.					
8	Help each other with work demands.					
9	Build respect for each other's particular interests.					
10	Get family members to carry out their responsibilities when they neglect them.					
11	Build trust in each other.					
12	Figure out what choices to make when the family faces important decisions.					
13	Find community resources and make good use of them for the family.					
14	Get the family to keep close ties to their larger family.					
15	Celebrate family traditions even in difficult times.					
16	Cooperate with schools to improve their educational practices.					
17	Face up to difficulties without excessive tension.					
18	Remain confident during difficult times.					
19	Accept each member's need for independence.					
20	Serve as a positive example for the community.					

Supplementary Table S2. Arabic version of The Perceived Collective Family Efficacy scale

كثيرا جدا	كثيرا	أحيانا	نادرا	أبدا	من خلال التعامل مع بعضكم البعض داخل الأسرة، إلى أي مدى يتمكن أفراد أسرتك من:	
					تخصيص وقت الفراغ للأسرة عندما تكون هناك أشياء أخرى تستدعي الاهتمام	1
					الموافقة على القرارات التي تتطلب بعض التضحية بالمصالح الشخصية	2
					حل النزاعات عندما يشعر أفراد الأسرة أنهم لا يعاملون بشكل عادل	3
					منع الخلافات الأسرية من التحول إلى جدال محتدم	4
					طلب مشاركة المسؤوليات المنزلية من أفراد الأسرة	5
					دعم بعضهم البعض في أوقات التوتر	6
					مساعدة بعضهم البعض لتحقيق أهدافهم الشخصية	7
					مساعدة بعضهم البعض في متطلبات العمل	8
					بناء احترام للمصالح الفردية لبعضهم البعض	9
					مطالبة أفراد الأسرة بالقيام بمسؤولياتهم عند إهمالهم لها	10
					بناء الثقة لبعضهم البعض	11
					تحديد الخيارات التي يجب عليهم اتخاذها عندما تواجه الأسرة قرارات مهمة	12
					البحث عن موارد المجتمع واستخدمها بشكل جيد للأسرة	13
					جعل الأسرة تحافظ على علاقات وثيقة مع أسرتها الممتدة	14
					الاحتفال بالمناسبات الأسرية حتى في الأوقات الصعبة	15
					التعاون مع المدارس لتحسين الممارسات التعليمية لفراد الأسرة	16
					مواجهة الصعوبات دون اجهاد مفرط	17
					المحافظة على الثقة في النفس خلال في الأوقات الصعبة	18
					تقبل حاجة كل عضو للاستقلال	19
					العمل على أن يكونوا قدوة إيجابية للمجتمع	20

Supplementary Table S3. Back translation version of The Perceived Collective Family Efficacy scale

	By dealing with each other in your family, to what extent can your family members:	Never	Rarely	Sometimes	Often	Very often
1	Allocate free time for the family when there are other things that require attention.					
2	Agree to decisions that require some sacrifice of personal interests.					
3	Resolve conflicts when other people feel like they are not being treated fairly.					
4	Prevent family disputes from mounting up to a heated conflict.					
5	Ask family members to participate in household responsibilities.					
6	Support each other in stressful times.					
7	Help each other with achieving personal goals.					
8	Help each other with work requirements.					
9	Build respect for each other's personal interests.					
10	Request family members to carry out their responsibilities when they neglect them.					
11	Build trust in each other.					
12	Determine choices to be taken when the family faces important decisions.					
13	Look up for community resources and use them for the wellbeing of the family.					
14	Help the family maintain close ties with the extended family.					
15	Celebrate family occasions even during hard times.					
16	Cooperate with schools in order to improve learning practices of family members.					
17	Face difficulties effortlessly.					
18	Maintain self-confidence even in difficult times.					
19	Accept each member's need for independency.					
20	Work on being a positive role model for the community.					
